# Prevalence, and early childhood caries risk indicators in preschool children in suburban Nigeria

**DOI:** 10.1186/s12903-015-0058-y

**Published:** 2015-06-30

**Authors:** Morenike O Folayan, Kikelomo A Kolawole, Elizabeth O Oziegbe, Titus Oyedele, Olusegun V Oshomoji, Nneka M Chukwumah, Nneka Onyejaka

**Affiliations:** Department of Child Dental Health, Obafemi Awolowo University, Ile-Ife, Nigeria; Oral Habit Study Group, Ile-Ife, Nigeria; Department of Child Dental Health, Obafemi Awolowo University Teaching Hospitals Complex, Ile-Ife, Nigeria

**Keywords:** Early childhood caries, Risk indicators, Age, Maternal knowledge, Sex, Prevalence

## Abstract

**Background:**

Early Childhood Caries (ECC) is defined as the presence of caries lesion in an primary tooth in children below the age of 71 months. It is a significant public health problem with consequences for the growth and development of affected children. The objective of this study was to determine the prevalence and ECC risk indicators in a suburban population in Nigeria.

**Methods:**

The data of 497 children aged 6 months to 71 months who were recruited through a household survey conducted in Ile-Ife, Nigeria was analysed for prevalence of ECC and risk indicators. Information on children’s ages, sex, socioeconomic status, tooth brushing habits, sugary snacks consumption, use of fluoridated toothpaste, birth rank, infant-feeding practices, breastfeeding practices, maternal age at childbirth, and maternal knowledge of oral health was obtained. Children’s oral hygiene and caries status was also determined. Risk factors associated with ECC were determined using logistic regression analysis.

**Results:**

Thirty-three (6.6 %) children had ECC. Four (0.8 %) had severe ECC. The four risk indicators for ECC were the child’s gender, mothers’ knowledge of oral health, consumption of sugary snacks in between meals more than three times a day, and the child’s oral hygiene status. Females (PR: −0.06; 95 % CI: −0.01– -0.01; p = 0.02), and children with mothers who had good knowledge of oral health (PR: −0.06; 95 % CI: −0.11––0.008; p = 0.02) were less likely to have ECC. Children who consumed sugary snacks in between meals three times a day or more (PR: 0.05; CI: 0.003 – 0.01; P = 0.04) and children with fair oral hygiene (PR: 0.05; 95 % CI: 0.005–0.10; p = 0.03) were more likely to have ECC.

**Conclusions:**

The prevalence of ECC in the study population was low. Promoting good oral hygiene practices and enhancingmothers’ knowledge of oral health may help reduce further, the risk for ECC in the study population.

## Background

Dental caries is a major public health concern. The most prevalent public health disease worldwide in 2010 was caries [1], with caries affecting the primary teeth being the tenth most prevalent disease [1]. Caries affecting the primary teeth in preschool children, also known as early childhood caries (ECC), has been of major concern in the field of child care. ECC is defined as one or more caries lesions, with or without cavitation, by the age of 71 months [2]. The prevalence of ECC is especially high in many low-income countries [3, 4] and in socioeconomically disadvantaged groups [5–7].

In many countries, ECC are largely untreated [8, 9]. Unfortunately, caries has major impact on children’s quality of life causing many to suffer pain, premature tooth-loss, malnutrition, and delayed growth and development [10, 11]. In addition, children with caries spend more time out of school than in school and do not engage actively in outdoor activities because of restrictions from caries-associated pain [12, 13]. ECC is also a risk factor for caries in the permanent dentition [14].

Many factors are associated with ECC. These include the presence of plaque, poor oral hygiene, increasing age, sex, and the frequency and timing of consumption of sugar-containing drinks [15–18]. Other reported associated factors are nocturnal breastfeeding [19] and prolonged duration of breastfeeding [20], but some authors have questioned this association [21, 22]. Other risk factors include the presence of enamel hypoplasia [23], molar-incisor hypomineralisation [24], and the child’s socioeconomic status [25]. Many maternal factors that may predispose children to ECC are children’s oral-hygiene behavior, infant-feeding practices [26], maternal knowledge of oral hygiene practices [27], maternal nutrition [28] and maternal stress [29].

One of the biggest challenges facing caries management in children in Nigeria is the poor uptake of treatment services for caries [30]. A combination of biological, behavioral, and structural interventions to prevent lesions and promote prompt diagnosis has been proposed [31]. However, research-based evidence is needed to aid the design of caries prevention and risk mitigation programs in Nigeria. While some research had identified risk factors for ECC in Nigeria, little is known about the interplay of these factors and how they affect the risk of ECC.

This study would determine the prevalence of ECC by conducting a secondary analysis of a data set collected through a household survey. We shall also determine which biological (age, sex, socioeconomic status, birth rank, family size, family structure, maternal age at childbirth) and non-biological (oral health knowledge of mothers, infant feeding practice, tooth brushing frequency, use of fluoridated toothpaste, frequency of sugar consumption in-between meals, oral health status) factors are ECC risk indicators for the study population.

## Methods

### Study design

This report is the outcome of a secondary data analysis of a cross-sectional study conducted in the Ife-Central Local Government Area (LGA), a suburban area. The primary study looked at the association between digit sucking and the prevalence of caries in the study population. Part of this main study has been reported in a study by Folayan, et al. [32]. Data were collected from 992 children and parents of children aged 6 months to 12 years old through a household survey.

### Sampling procedure

The sampling was done using a (three-level) multi-stage cluster sampling aimed at selecting eligible study participants. Stage 1 involved the random selection of eight out of the 25 enumeration areas within Ife Central LGA designated by the National Populations Commission during the 2006 National census exercise by balloting. Stage 2 involved the selection of eligible households within the enumeration sites for the survey. At each of the enumeration sites, every third household on each street was considered eligible for recruitment of a study participant. Stage 3 involved the selection of actual respondents for interview and examination. Only one r each household participated in the study. Alternative sexes and age range identified for study recruitment were selected to participate in each consecutive household. Study participant recruitment continued in the enumeration site until the study sample per each data collector was reached.

### Study population

Ife Central Local Government Area has an estimated population of 167,204 in the year 2006 [32]. The data of a subset of children aged 6 months to 71 months were analyzed for this study. The study population consisted of children living with their biological parents or legal guardians and for whom the parents or legal guardians gave written informed consent for their child to participate in the study. Only children who were present in the home at the time the study was conducted were recruited for the study.

The primary study proposed to exclude from analysis, the data of children with chronic medical conditions that required prolonged use of sweetened medication, antihistamines, and anti-asthmatic drugs; those with medical conditions that increased the risk for caries, such as Sicca syndrome or Sjögren’s syndrome, or other conditions associated with xerostomia; and those with dental developmental anomalies, such as deciduous molar hypoplasia, that result in defective enamel formation and increase risk for caries. However, the data of these children were included in the current study analysis.

### Study size

Using the formula by Araoye [33], we calculated that the sample size required to determine the prevalence of ECC in the study population was 144 participants, using an ECC prevalence of 10.5 % [34], a margin of error of 5 % and a confidence level of 95 %. However, our study population included the data of 497 children age 6 months to 71 months retrieved from the primary data source.

### Data collection

Data were collected through personal interviews by use of a structured questionnaire. Experienced fieldworkers who had been engaged in past national surveys and who were trained for this study administered the study tool in the field. Four trained qualified dentists were engaged to do the clinical examinations for the study participants. The dentists were trained centrally for the purpose of this study. They had several sessions reviewing clinical photographs and repeated practices on examination of lesions using clinical photographs until their competency to make diagnosis was as close to perfect with that of the training consultants. Clinical examination was conducted on 10 patients with recordings made for caries, oral health status, and gingival health. The same patients were re-examined two weeks later. The kappa scores for the dentists ranged between 0.7-1.0.

The instruments were administered to the mothers when both parents were at home. When only one parent was met at home during the survey, the questionnaire was administered to that parent. Data collected during the survey included the socio-demographic profile. Questions on oral health knowledge of mothers and oral health behavior of children and infant-feeding profile were collected from the child’s mother. Intra-oral examination was conducted to determine the caries and oral hygiene status.

#### Socio-demographic profile

Information on the age, sex, family composition, birth rank, family size, maternal age at childbirth, and socioeconomic status of the children was collected. The age was established as the child’s age at their last birthday. Sex was determined as male or female. The birth rank of the child was determined as the birth position of the child among his or her siblings. The family composition of the child was also recorded: child could be living with both parents, with mother only, with father only, with mother/father and step parent or with the caregiver.

Data on socioeconomic status were determined by use of an adapted version of the index developed by Olusanya et al. [35], which had been used a previous study in the study environment [36]. The index is a multiple-item index combining the mother’s level of education with the father’s educational level and occupation. For this study, data were collected on the educational levels and professions of respondents’ parents. The mother’s level of education was classified as follows: no formal education, Quranic and primary school education (score 2); secondary school education (score 1) and tertiary education (scored 0). The father’s occupation was also categorized into three levels: civil servants or skilled professionals with a tertiary level of education (score 1); civil servants or skilled professionals with a secondary level of education (score 2); unskilled, unemployed, students, and civil servants or skilled professionals with a primary and or Quranic education (scored 3}. The social class of the parents was determined by adding the score of the mother’s level of education to that of the father’s occupation. Each child was allocated into social classes I–V (class I, upper class; class II, upper middle class; class III, middle class; class IV, lower middle class; class V, lower class). When a child had lost a parent, their socioeconomic status was determined using the status of the living parent.

#### Oral health knowledge

The process for assessing parents’ oral health knowledge for this study population had been described in detail in a prior study [32]. For this study, we used the data collected on the oral health knowledge of the children’s mothers. Respondents were asked to react to eight statements about aspects of caries diagnosis and prevention on a five-point Likert scale ranging from “strongly agree” to “agree”, “disagree”, “strongly disagree”, and “do not know”. The statements were: (i) Fluoridation of drinking water is an effective, safe, and efficient way to prevent dental caries. (ii) Use of fluoride-containing toothpaste is an effective, safe, and efficient way to prevent holes from forming on the teeth. (iii) Frequency of sugar consumption has a greater role in producing caries than the total amount of sugar. (iv) Sealant is effective in the prevention of pit and fissure caries in newly erupted molars. (v) Rinsing teeth with a little amount of water after brushing teeth increases the effect of fluoride. (vi) Using fluoride toothpaste is more important than the brushing per se for preventing caries. (vii) Brushing twice daily with fluoride-containing toothpaste is effective for preventing holes from developing in the teeth. (viii) It is important to visit the dental clinic regularly as a measure for preventing holes from forming in the teeth. For each of the eight statements, respondents who indicated “strongly agree” and “agree” as options were graded as having responded correctly to the statement. The responses were then scored from one to five with “strongly agree” scoring 5 and “do not know” scoring 1. Where there were no responses, a score of 1 was allocated. Therefore, each respondent could obtain a total minimum score of 8 and a total maximum score of 40. Mothers who scored 21 and above were categorized as having good oral health knowledge while those who scored 20 and below were categorized as having poor oral health knowledge [32].

#### Oral health behavior

The process for assessing the oral health behavior in this study population had also been described in detail in a previous study [32]. Information was generated on the tooth brushing frequency, use of fluoridated toothpaste, and eating sugary snacks between main meals for each of the children recruited for the study. These questions had four to seven alternatives. To define acceptable levels for each of the components, the following cutoff points were used: brushing more than once a day, using fluoridated toothpaste always or almost always, and eating sugary snacks between main meals less frequently than once a day.

The respondents were also asked to indicate the time of their last check-up (with the alternatives being within the last 6 months, more than 6 months to 1 year ago, more than 1 to 2 years ago, more than 2 to 5 years ago, more than 5 years, never, or do not remember). Attending a dental check-up within the last 12 months was defined as the use of preventive care.

Recommended oral self-care was defined as a composite score derived from indications of brushing teeth more than once a day, use of fluoridated toothpaste, and consumption of sugary snacks between main meals less frequently than once a day [32]. Each respondent had to have met these three criteria to be categorized as practicing the recommended oral self-care.

#### Infant-feeding profile

Mothers of the respondents were questioned on the form and duration of breastfeeding, and night-feeding patterns. Breastfeeding was classified as exclusive when the mother gave only breast milk without any other supplements for the first 6 months of life [37]; almost exclusive when the mother fed the child on breast milk with water supplements; and partial or mixed breastfeeding when other types of feeding were included with the breastfeeding. Night-feeding practice was defined as feeding the child at nighttime after going to bed.

#### Caries assessment

ECC was defined as dental caries in the primary teeth of children 5 years or younger [38]. Severe ECC (S-ECC) was defined as any sign of smooth-surface caries in children younger than 3 years of age; one or more decayed, missing or filled smooth-surface caries in primary maxillary anterior teeth in children 3–5 years old; or one or more decayed, missing or filled tooth greater or equal to 4 (for children 3 years of age), or to 5 (for children 4 years of age) or to 6 (for children 5 years of age) surfaces [38].

For this study, the number of decayed, filled, or missing teeth (dmft) was noted for each child who had caries. The dmft was determined according to the World Health Organization Oral Health Survey methods [39]. The examination for dental caries was conducted by use of a plain mouth mirror with a light source from a torch with the child seated on a chair. Teeth were not dried before examination, but gross debris was cleared with gauze where necessary. The examination of the teeth was done in an orderly manner from one tooth or tooth space to the adjacent tooth or tooth space.

To arrive at a dmft score for an individual child, three values were determined: the number of teeth with carious lesions, the number of teeth extracted due to caries, and the number of teeth with fillings or crowns [40]. The children’s parents were asked to explain the loss of any teeth not noted during the oral examination. Only teeth extracted due to caries were recorded as missing. The number of teeth was counted to give the dmft score for the primary dentition for each child. For the purpose of analysis, caries was classified as being present or not present.

#### Oral hygiene status

Mouth cleanliness was evaluated by assessing the accumulation of plaque and debris. The Simplified Oral Hygiene Index (OHI-S) of Greene and Vermillion [41] was used to determine the oral hygiene status. The OHI-S comprises debris and calculus scores on selected tooth surfaces. However, because of the young age group involved, facial and lingual surfaces were examined on the following index teeth 51, 55, 65, 71, 75, and 85. The debris and calculus scores were added and divided by the number of surfaces examined to give the OHI-S score as recommended. Oral hygiene was classified as good, fair, or poor when the score ranges were 0.0–1.2, 1.3–3.0, and >3.0, respectively.

### Theoretical Model for statistical analysis

**Fig. 1:**
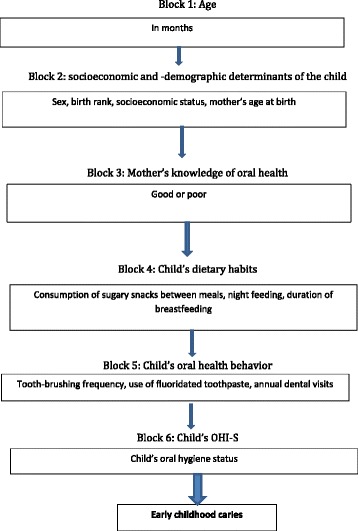
Hierarchical model for data analysis

The theoretical model used by Nunes et al. [42] was adapted for use in this study. A hierarchical theoretical model with the following six blocks was employed: 1) age of child; 2) socioeconomic and demographic factors; 3) mother’s knowledge of oral health; 4) child’s dietary child habits; 5) child oral hygiene data; and 6) oral hygiene index. See Fig. 1.

Age was considered a potentially confounding factor, so the developed model was adjusted for this variable. The second block included socioeconomic and demographic variables as distal factors in the theoretical model since they can influence all variables in subsequent blocks. Also included in this block were the child’s birth rank and the mother’s age in years and the family size. The health variable included for the mother in the third block, which may mediate the association between socioeconomic variables and childhood caries, was mother’s knowledge of oral health. In the fourth block, dietary practices were included, assuming that these variables may be influenced by socioeconomic and demographic factors, and by mother’s knowledge of oral health variables. The variables in this block were: nighttime breastfeeding (yes, no), prolonged breastfeeding beyond 12 months (yes, no), and frequency of consumption of sugary snacks between main meals (<3 or ≥3 times/day). Oral hygiene practice may be moderator variable of the association between diet and ECC, so it was included in the fifth block as the number of times a day the child’s teeth are brushed (up to 1 time, ≥2 times) and the daily use of fluoridated toothpaste. In the sixth block, the oral hygiene status was recorded with the assumption that this variable may be influenced by both hygiene and dietary practices.

### Data analysis

#### Data transformation

For ease of analysis, socioeconomic status in this study was regrouped into three levels: high (upper and upper middle classes), middle (middle class), and low (lower middle and lower classes). This categorization was used to test associations and for logistic regression analysis. This modality of categorization of socioeconomic status was previously used by Folayan et al. (36).

The age of the children was categorized into <12 months, 12–23 months, 24–35 months and 36–47 month, 48–59 months and 60–71 months based on the recommendation by Dury et al. [38].

Each of the following oral health behaviors was identified as present if tooth brushing was done more than once a day, fluoridated toothpaste was used always or almost always, and when sugary snacks were eaten between main meals less frequently than once a day. A child was categorized as using recommended oral self-care measures when all three behaviors were present.

Further sub-analysis was conducted for children who consumed sugary snacks were eaten between main meals. Based on the outcome by Folayan et al. [43], the caries prevalence of children who consumed sugary snacks were eaten between main meals less than three times a day was compared with that of children who consumed sugary snacks were eaten between main meals three times a day or more.

Maternal age at childbirth was dichotomized to 22 years and younger or above 22 years. The age of 22 years was chosen as the age for dichotomization based on the study by Niji et al. [44]. Family size was also dichotomized to up to four siblings or more than four siblings. Having four siblings was used as the reference point for dichotomization because the official national standard family size for Nigeria is four children. The family composition of each child was also dichotomized: child could either be living with both parents or with others (mother only, with father only, with mother/father and step parent or with guardian). The birth rank was dichotomized into ‘Primogenitor or only child’ or ‘Not primogenitor’ based on an earlier classification by Ola et al. [45].

#### Data analysis

Descriptive analysis was conducted to determine the prevalence of ECC in the study population for each age, each sex, and each socioeconomic stratum. The chi-square test was used to test associations between the ECC and (i) the child’s oral health behavior, (ii) the mother’s oral health knowledge, (iii) infant-feeding practices, (iv) age, (v) socioeconomic status, (vi) sex, (vii) oral hygiene status, and (viii) recommended oral self-care was tested.

A logistic regression using forward selection and guided by a hierarchical approach as described was used for the inferential analysis. We evaluated possible associations between risk factors and ECC using a series of models as described in the section on theoretical model. However, only factors whose p value was <0.20 during the tests of association entered into the model based on recommendation by Altman [46].

The hierarchical modeling started with the first block. The variables of the first block were adjusted simultaneously for each other and only those variables whose p value was <0.20 entered in subsequent models. Then variables of the second block were adjusted simultaneously for each other and for the variables whose p value was <0.20 in the previous step. The significance of each variable was considered at the time of entry into the model (p value <0.05). All other blocks were then added in succession following the same procedure. The estimated coefficients were expressed as prevalence ratios (PRs) and their 95 % confidence intervals were also calculated. Each model was tested for the goodness of fit. All variables were assessed for their normalcy of distribution. Where data were skewed, the data either squared to adjust for their skewness or their dichotomized version used in the model. STATA software (version 10) was used for data processing and statistical analysis.

### Ethical consideration

Before commencing the study, ethical approval for the study was obtained from the Health Research Ethics Committee of the Obafemi Awolowo University Teaching Hospitals’ Complex Ile-Ife (ERC/2013/07/14). Permission to conduct the study was also obtained from the Ife Central Local Government. Efforts were made to ensure confidentiality and adherence to ethical principles during fieldwork. All data were collected without study participants’ identifiers (names and residential address). All study participants were compensated for their time with gifts worth less than $1.00.

## Results

Thirty-three (6.6 %) of the 497 children examined had ECC and four (0.8 %) had severe ECC. The prevalence of ECC in children 6–11 months, 12–23 months, 24–35 months, 36–47 months, 48–59 months, 60-71months were 0.0 %, 1.6 %, 2.1 %, 8.2 %, 12.7 %, and 6.6 %, respectively. Seventy two carious teeth identified, of which 67 (93.1 %) were unrestored decayed teeth and five (6.9 %) were extracted teeth. None of the teeth was restored. The dmft ranged from 0 to 8. Four hundred sixty-four (93.4 %) children were caries-free, 11 (2.2 %) had a dmft of 1, 13 (2.6 %) had a dmft of 2, three (0.6 %) had a dmft of 3, and one (0.2 %) had a dmft of 4, 5, and 6, respectively. Two children had a dmft of 8 (0.4 %). The mean dmft was 0.15. Table 1 shows the caries profile of children with ECC.

**Table 1:** Distribution of children with ECC and severe ECC

Age	ECC	S-ECC	Mean dmft	Total no of children with ECC	Total no of children
6-11 months	0 (0.0 %)	0 (0.0 %)	0	0 (0.0 %)	10 (2.0 %)
12-23 months	0 (0.0 %)	1 (25.0 %)	0.13	1 (3.0 %)	62 (12.5 %)
24-35 months	2 (6.9 %)	0 (0.0 %)	0.02	2 (6.1 %)	95 (19.1 %)
36-47 months	10 (34.5 %)	0 (0.0 %)	0.14	10 (30.3 %)	122 (24.6 %)
48-59 months	11 (37.9 %)	2 (50.0 %)	0.27	13 (39.4 %)	102 (20.5 %)
60-71 months	6 (20.7 %)	1 (25.0 %)	0.21	7 (21.2 %)	106 (21.3 %)
Total	29 (100.0 %)	4 (100.0 %)	0.15	33 (100.0 %)	497 (100.0 %)

Table 2 shows the profile of the study participants. It also shows the outcome of the tests of association between ECC and the child’s oral health behavior, use of recommended oral self-care, infant-feeding practices, age, socioeconomic status, sex, birth rank, family size and oral hygiene status. Also tested was the association between ECC and the mother’s oral health knowledge and maternal age at child birth.

**Table 2:** Association between ECC, socio-demographic profile, oral health practices, mother’ oral health knowledge, oral hygiene, and infant-feeding practices

Variables	ECC present n = 33	ECC absent n = 464	Total N = 497	P value
**Age**				
6-11 months	0 (0.0 %)	10 (2.2 %)	10 (2.0 %)	0.01
12-23 months	1 (3.0 %)	61 (13.1 %)	62 (12.5 %)	
24-35 months	2 (6.1 %)	93 (20.0 %)	95 (19.1 %)	
36-47 months	10 (30.3 %)	112 (24.1 %)	122 (24.6 %)	
48-59 months	13 (39.4 %)	89 (19.2 %)	102 (20.5 %)	
60-71 months	7 (21.2 %)	99 (21.3 %)	106 (21.3 %)	
**Sex**				
Male	11 (33.3 %)	255 (55.0 %)	266 (53.5 %)	0.02
Female	22 (66.7 %)	209 (45.0 %)	231 (46.5 %)	
*Socioeconomic status				
High	5 (15.2 %)	124 (26.7 %)	129 (26.0 %)	0.07
Middle	13 (39.4 %)	213 (45.9 %)	226 (45.5 %)	
Low	15 (45.5 %)	127 (27.4 %)	142 (28.5 %)	
^*^ **Maternal age at childbirth**				
>22 years	5 (15.6 %)	90 (21.3 %)	95 (20.9 %)	0.45
≤22 years	27(84.4 %)	332 (78.7 %)	359 (79.1 %)	
^*^43 variables missing				
*Birth rank				
Primogenitor or only child	11 (33.3 %)	234 (51.0 %)	245 (49.8 %)	0.05
Not primogenitor	22 (66.7 %)	225 (49.0 %)	247 (50.2 %)	
*5 variables missing				
*Family size				
More than four siblings	2 (6.1 %)	20 (4.3 %)	22 (4.4 %)	0.65
Four siblings or less	31 (93.9 %)	442 (95.7)	473 (95.6 %)	
*2 variables missing				
*Family composition				
Living with both parents	27 (81.8 %)	421 (92.3 %)	448 (91.6 %)	0.30
Living with others	6 (18.2 %)	35 (7.6 %)	41 (8.4 %)	
P = 0.03 *8 variables missing				
**Tooth brushing frequency**				
Brushing teeth twice daily or more	3 (9.1 %)	40 (8.9 %)	43 (8.9 %)	0.76
Brushing teeth less than twice daily	30(90.9 %)	424 (91.1 %)	454 (81.1 %)	
**Use of fluoridated toothpaste**				
Use fluoridated toothpaste	33 (100.0 %)	438 (94.4 %)	471 (94.8 %)	0.40
Do not use fluoridated toothpaste	0 (0.0 %)	26 (5.6 %)	26 (5.2 %)	
*Consume sugary snacks between meals				
Consume sugary snacks between meals three times a day or more	16 (50.0 %)	130 (29.2 %)	146 (30.6 %)	0.01
Consume sugary snacks between meals less than three times a day	16 (50.0 %)	312 (70.8 %)	331 (69.4 %)	
*20 variables missing				
**Recommended oral self care**				
Use recommended oral self-care	0 (0.0 %)	5 (1.1 %)	5 (1.0 %)	1.00
Do not use recommended oral self-care	30 (100.0 %)	459 (98.9 %)	492 (99.0 %)	
**Annual dental service utilization**				
Had dental checkup last 12 months	2 (6.1 %)	17 (3.7 %)	19 (3.8 %)	0.49
Did not have dental checkup in last 12 months	31 (93.9 %)	447 (96.3 %)	478 (96.2 %)	
**Maternal oral health knowledge**				
Good	16 (48.5 %)	333 (71.8 %)	349 (70.2 %)	0.005
Poor	17 (51.5 %)	131 (28.2 %)	148 (29.8 %)	
*Infant-feeding practices				
Night breastfeeding	13 (39.4 %)	155 (33.4 %)	128 (25.8 %)	0.46
No night breastfeeding	20 (60.6 %)	309 (66.6 %)	369 (74.2 %)	
*P* = 0.46				
Exclusive breastfeeding	5 (15.6 %)	52 (11.2 %)	57 (11.5 %)	0.84
Partially exclusive breastfeeding	1 (3.0 %)	14 (3.0 %)	15 (3.0 %)	
Nonexclusive breastfeeding	26 (78.8 %)	342 (73.7 %)	368 (74.0 %)	
*P* = 0.84 *56 variables missing				
Breastfed for ≤12 months	10 (31.3 %)	91 (22.0 %)	101 (22.7 %)	0.23
Breastfed for >12 months	22 (68.7 %)	322 (78.0 %)	344 (77.3 %)	
*52 variables missing				
*Oral hygiene status				
Good	19 (57.6 %)	324 (70.4 %)	343 (69.6 %)	0.18
Fair	14 (42.4 %)	125 (27.2 %)	139 (28.2 %)	
Poor	0 (0.0 %)	11 (2.4 %)	11 (2.2 %)	

*4 variables missing

The age and sex of the child, and mother’s oral health knowledge were significantly associated with presence of ECC. The proportion of children with caries increased significantly with increasing age up to the age of 4 years (p = 0.01). More females than males had ECC (66.7 % vs 33.3 %; p = 0.02). More children with mothers who had good oral health knowledge were ECC free when compared with children who had mothers with poor oral health knowledge (71.8 % vs 28.2 %; p = 0.005).

The child’s birth rank was also significantly associated with having ECC. More children who were not progenitors or only child had ECC when compared with children who were progenitors or only child (33.3 % vs 66.7 %; p = 0.05).

Also, more children who consumed cariogenic snacks between meals three times a day or more were ECC free compared with children who consumed cariogenic snacks in-between meals less than three times a day (70.8 % vs 29.2 %; p = 0.01).

There was no significant difference in the proportion of children with high, middle and low socio-economic status who had ECC (p = 0.07). Maternal age at childbirth (p = 0.45), birth rank (p = 0.06), family size (p = 0.65), family composition (p = 0.30), brushing teeth twice daily (p = 0.76), use of fluoridated toothpaste (p = 0.40), annual dental visits (p = 0.49) and the oral hygiene status (p = 0.18) were not significantly associated with ECC. The use of a combination of caries prevention tools (recommended oral self-care measures) was also not associated with ECC (p = 1.00). Also, no infant-feeding practice was significantly associated with ECC: night breastfeeding (p = 0.46), forms of breastfeeding (exclusive, partial, or nonexclusive) (p = 0.84), and duration of breastfeeding (p = 0.23) were all not associated with ECC.

Table 3 shows the results from the logistic regression analysis determining the risk indicators for ECC. Analysis could only be conducted for blocks 1–4 and 6 since none of the variables in block 5 reached a significant value of p < 0.20 for the tests of association. The logistic regression analysis showed four risk indicators for ECC: gender, mothers’ knowledge of oral health, consumption of sugary snacks between meals and oral hygiene status. Females were less likely than males to have ECC (PR: −0.06; 95 % CI: −0.01– -0.01; p = 0.02). Children with mothers who had good knowledge of oral health were less likely than children with mothers who had poor knowledge of oral health to have ECC (PR: −0.06; 95 % CI: −0.11––0.008; p = 0.02). Children who consumed sugary snacks in between meals three times a day or more were more likely than children who consumed sugary snacks in between meals less than three times a day to have ECC (PR: 0.05; CI:0.003 – 0.01; P = 0.04). Children with fair oral hygiene were more likely to have ECC than children with good oral hygiene (PR: 0.05; 95 % CI: 0.005–0.10; p = 0.03).

**Table 3:** Logistic regression determining risk indicators for early childhood caries

Variables	Block 1PR (95 % CI)	P value	Block 2PR (95 % CI)	P value	Block 3PR (95 % CI)	P value	Block 4PR (95 % CI)	P value	Block 6PR (95 % CI)	P value
*Age*	0.02(0.003-0.04)	0.02	0.02(0.00-0.03)	0.04	0.02(0.001-0.03)	0.03	0.02(−0.00 – 0.03)	0.06		
*Sex*										
Male			-	**-**	-	-	-	-	-	-
Female			−0.05(−0.10- -0.009)	0.02	−0.05(−0.09- -0.005)	0.03	−0.05(−0.10- -0.008)	0.02	−0.06(−0.10- -0.01)	0.02
*Socioeconomic status*										
High			-	-						
Middle			−0.04(−0.02-0.10)	0.20						
Low			−0.02(−0.07-0.02)	0.50						
*Birth rank*										
Not primogenitor			-	-						
Primogenitor or only child			−0.04(−0.09—0.005)	0.08						
*Mothers’ knowledge of oral health*										
Good					-	-	-	-	-	-
Poor					−0.07(−0.11 - -0.02)	0.007	−0.05(−0.10- -0.002)	0.04	−0.06(−0.11- -0.008)	0.02
*Sugar consumption habit between meals*										
≥3 times a day							-		-	-
<3 times a day							0.05(−0.000- 0.10)	0.05	0.05(0.003 -0.10)	0.04
*Oral hygiene status*										
Good									-	-
Fair									0.05(0.005-0.10)	0.03
Poor									−0.05(−0.20-0.10)	0.53

## Discussion

The study showed that the prevalence of ECC in the study population was low. The risk of ECC was significantly higher for children with mothers who had poor knowledge of oral health, males, children with fair oral hygiene and those who consumed sugary snacks in between meals more than 3 times a day.

One of the strengths of this study is that the data were collected through a household survey, which means that the results of the study are generalizable to the study population. A prior study on ECC in the study environment was hospital based [43] thus limiting the generalizability of the study findings. Another strength of this study is the inclusion of various potential risk variables in the multivariate regression model predicting ECC for the study population which differ from those studies that had simply conducted a univariate analysis [43] and had evaluated limited potential risk factors for ECC [47]. Also, the use of a theoretical approach for statistical analysis reduced the risk for spurious outcomes.

This study was also able to show that oral hygiene status was a significant risk factor for ECC like the study conducted Sowole et al. [47]. However, unlike Sowole et al. [47] and Folayan et al. [43], this study identified gender as a risk factor for ECC. The plausibility of biological factor like sex being risk factors for dental caries is difficult to understand and may require further studies to investigate gender differences in caries risk practices or differences in tooth anatomy between sexes in this study location. The differences in the socialization process of males and females in the study environment may also need to be studied to be able to find appropriate reasons for the observation.

This study, like past studies [48, 49], was also able to show that consumption of sugary snacks between meals was a risk factors for ECC. The finding also corroborates an earlier finding in the study environment that consumption of sugary snacks between meals more than three times a day was a risk factor for ECC [43]. This further reinforces the need to manage children with this characteristic as having a high risk for ECC. The relationship between diet and dental caries has however become weaker in contemporary society attributable to the widespread use of fluoride.

The study was however, unable to show that the other caries prevention practices (use of fluoridated toothpaste, brushing twice a day and annual dental visits) could mitigate the risk of ECC. This finding does not corroborate the findings of a previous study that the use of a combination of fluoridated toothpaste and twice daily tooth brushing was the most effective caries prevention measure for children in the study environment [50]. While this study only looked at the preschool children and caries in the primary teeth, the previous study examined caries in a wider age range which included children with permanent dentition. The difference observed may therefore be a difference in the impact of fluoridated toothpaste and twice daily tooth brushing on caries in the primary and permanent dentition. There is clear evidence that regular use of fluoride toothpastes have a caries inhibiting effect in the permanent dentition but little evidence on its impact in the primary dentition [51]. These findings should however, not dissuade children less than six years of age from the use of fluoridated toothpastes and twice daily brushing, as good oral health habits need to be developed from infancy. Twice daily brushing with fluoridated toothpastes is not only important for ensuring continued availability of fluoride in the oral environment; when done effectively it reduces plaque accumulation.

The prevalence of ECC in this study populationwas low when compared with the prevalence of 28 % in the United States [52], 32 % for 3–4-year-old children in Greater Manchester, UK [53], 56.2 % in 3-year-old Polish children [54], 50 % to 80 % in high-risk populations in Canada [55, 56], and as high as 70 % in socially disadvantaged groups in Europe, Africa, Asia, the Middle East, and North America [57]. Very little is understood about the reason for the low ECC prevalence in Nigeria. This may reflect the reason for the very low DMFT in 12 year olds and adults in the country [58]. It would be important to conduct studies that can help identify reasons for the low prevalence of ECC observed in the study population. This would enable policy makers to identify caries-prevention practices that need to be reinforced in the study population. It may also help the global community learn about factors that could reduce the risk of ECC.

The proportion of children with untreated caries was very high in this study. Children with untreated caries run the risk of poor quality of life and other short-term, long-term, and rare sequelae of dental caries [59]. The use of dental services is also low in the study population [60]. For this community with a low prevalence of ECC but high risk for untreated caries and the development of its sequelae, it may be important to conduct screening programmes adopting appropriate approaches for behavior change as posited by Folayan et al. [31].

This study has a number of limitations. First, the determination of ECC prevalence for the study population was based on data that identified caries by use of the WHO criteria. This would result in an underestimation of the prevalence of caries in the study population, as non-cavitated lesions are more prevalent on the smooth tooth surfaces of primary teeth in children aged 6–18 months than are cavitated lesions [38]. A primary study determining ECC should ideally use the ICDAS [61] for caries detection rather than the WHO criteria. Second, the data for this study were not collected primarily to address the objectives of this study. Though this secondary data analysis was powered to determine the prevalence of ECC, it was not powered to analyze factors associated with ECC in children below 72 months; thus the ability of the study to identify predictors of ECC was limited. However the conduct of a logistic regression analysis using a hierarchical data analysis model based on a theoretical framework helped us evaluate mediation of more proximal factors and their association with ECC [42], reduced the tendency for spurious associations and increased the dependability of the data analysis outcomes. Third, respondents had to recall feeding practices including recall of the duration of breastfeeding for the study participants. The reliability and validity of such recall data is high for the first 36 months and decreases after that [62]. The questionnaire assessing oral health knowledge of the mother also used ‘hole’ alternatively with ‘caries’ showing a lack of consistency in the wordings of the questionnaire with the possibility of introducing distortions in the responses.

Despite these limitations, this study once again reiterates the importance of maternal factors in the management of caries. Mothers with good oral health knowledge protect children from ECC. Unfortunately, while programmes can be designed to influence maternal oral health knowledge as a measure of improving oral hygiene practices and reducing consumption of sugary snacks between meals by children [32], little can be done to modify the biological factor (gender) that increases the risk of ECC for children in this study population. Further studies are required to understand how gender interplays with environmental factors to increase the risk of ECC for children in the study location.

For this population, not only is it important to promptly identify ECC, it is also important to identify ways that children with ECC can promptly access treatment because the level of untreated caries is extremely high. Prompt diagnosis and treatment of the lesion reduces the risk of developing new caries lesions. Prior studies show the risk for developing new caries lesions is five times higher in children with untreated caries lesions than in children with no caries [63]. The low prevalence of ECC in the study environment when compared with ECC prevalence in other countries around the world should not preclude efforts at prevention, prompt diagnosis, and early treatment of ECC in the study population.

## Conclusions

The prevalence of ECC in the study population is low, although the report may have underestimated the true prevalence of ECC in the community. Programs that improve the oral health knowledge of mothers should be able to result in improved oral hygiene practices and reduced consumption of sugary snacks between meals by preschool children. A study primarily designed to assess predictors of ECC in the preschool children in the study environment is required.

## Abbreviations

ECC: Early childhood caries
UK: United Kingdom

## References

[1] Marcenes W, Kassebaum NJ, Bernabe E, Flaxman A, Naghavi M, Lopez A (2013). Global burden of oral conditions in 1990–2010: A systematic analysis. J Dent Res.

[2] American Academy of Paediatric Dentistry: Policy on Early Childhood Caries (ECC): Classification, consequences and preventive strategies. 2011. Reference Manual 2011. Retrieved 14^th^ March 2014 from: http://www.aapd.org/media/Policies_Guidelines/P_ECCclassifications.pdf.

[3] Resini SD, Douglass JM (1998). Psychosocial and behavioral issues in early childhood caries. Community Dent Oral Epidemiol.

[4] Prakash P, Subramania MP, Durgesh BH, Konde S (2012). Prevalence of early childhood caries and associated risk factors in preschool children of urban Bangalore, India: A cross-sectional study. Eur J Dent.

[5] Stecksén-Blicks C, Borssén E (1999). Dental caries, sugar-eating habits and toothbrushing in groups of 4-year-old children 1967–1997 in the city of Umea, Sweden. Caries Res.

[6] Tomar SL, Reeves AF (2009). Changes in the oral health of US children and adolescents and dental public health infrastructure since the release of the Healthy People 2010 Objectives. Acad Pediatr.

[7] Hallet KB, O’Rourke PK (2006). Pattern and severity of early childhood caries. Community Dent Oral Epidemiol.

[8] Mehta A, Bhalla S (2014). Assessing consequences of untreated carious lesions using pufa index among 5–6 years old school children in an urban Indian population. Indian J Dent Res.

[9] Kassebaum NJ, Bernabé E, Dahiya M, Bhandari B, Murray CJ, Marcenes W: Global Burden of Untreated Caries: A Systematic Review and Metaregression. J Dent Res. 2015;94:650-8. 10.1177/0022034515573272 25740856

[10] Acs G, Lodolini G, Kaminski S, Cisneros GJ (1992). Effect of nursing caries on body weight in pediatric population. Pediatr Dent.

[11] Clarke M, Locker D, Berall G, Pencharz P, Kenny DJ, Judd P (2006). Malnutrition in a population of young children with severe early childhood caries. Paediatr Dent..

[12] Sheiham A (2006). Dental caries affects body weight, growth and quality of life in pre-school children. Br Dent J.

[13] Ginsburg KR (2007). American Academy of Pediatrics Committee on Communications; American Academy of Pediatrics Committee on Psychosocial Aspects of Child and Family Health. The importance of play in promoting health child development and maintaining strong parent–child bonds. Pediatrics..

[14] Li Y, Wang W (2002). Predicting caries in permanent teeth from caries in primary teeth: an eight-year cohort study. J Dent Res.

[15] Benjamin R (2010). Oral health, the silent epidemic. Pub Health Rep..

[16] Reisine S, Douglass JM (1998). Psychosocial and behavioural issues in early childhood caries. Community Dent Oral Epidemiol..

[17] Tinanoff N (1998). Introduction to early childhood caries conference: initial description and current understanding. Community Dent Oral Epidemiol..

[18] Declerck D, Leroy R, Martens L, Lesaffre E, Garcia-Zattera MJ, Broucke VS (2008). Factors associated with prevalence and severity of caries experience in preschool children. Community Dental Oral Epidemiol.

[19] van Palenstein Helderman WH, Soe W, van 't Hof MA (2006). Risk factors of early childhood caries in a Southeast Asian population. J Dent Res..

[20] Folayan MO, Sowole CA, Owotade FJ, Sote E (2010). Impact of infant feeding practices on caries experience of preschool children. J Clin Pediatr Dent..

[21] Iida H, Auinger P, Billings RJ, Weitzman M (2007). Association between infant breastfeeding and early childhood caries in the United States. Pediatrics.

[22] Masumo R, Bardsen A, Mashoto K, Åstrøm AN (2012). Prevalence and socio-behavioral influence of early childhood caries, ECC, and feeding habits among 6-36months old children in Uganda and Tanzania. BMC Oral Health..

[23] Oliveira AF, Chaves AM, Rosenblatt A (2006). The influence of enamel defects on the development of early childhood caries in a population with low socioeconomic status: a longitudinal study. Caries Res.

[24] Elfrink ME, Schuller AA, Veerkamp JS, Poorterman JH, Moll HA, ten Cate BJ (2010). Factors increasing the caries risk of second primary molars in 5-year-old Dutch children. Int J Paediatr Dent.

[25] Hallett KB, O'Rourke PK (2003). Social and behavioural determinants of early childhood caries. Aust Dent J..

[26] Finlayson TL, Siefert K, Ismail AI, Sohn W (2007). Maternal self-efficacy and 1-5-year-old children's brushing habits. Community Dent Oral Epidemiol..

[27] Leong PM, Gussy MG, Barrow SY, de Silva-Sanigorski A, Waters E (2013). A systematic review of risk factors during first year of life for early childhood caries. Int J Paediatr Dent..

[28] Moynihan PJ, Holt RD (1996). The national diet and nutrition survey of 1.5 to 4.5 year old children: summary of the findings of the dental survey. British Dental Journal.

[29] Finlayson TL, Siefert K, Ismail AI, Sohn W (2007). Psychosocial factors and early childhood caries among low-income African-American children in Detroit. Community Dent Oral Epidemiol..

[30] Folayan MO, Adeniyi AA, Chukwumah N, Onyejaka N, Esan A, Sofola OO (2014). Programme guidelines for promoting good oral health for children in Nigeria: a position paper. BMC Oral Health.

[31] Folayan MO, Chukumah NM, Onyejeka N, Adeniyi A, Olatosi O (2014). Appraisal of the national response to caries epidemic in children in Nigeria. BMC Oral Health.

[32] Folayan MO, Kolawole KA, Oyedele T, Chukumah NM, Onyejaka N, Agbaje H (2014). Oziegbe EO. Oshomoji OV: Association between preventive oral health habits of parents and caries experience in children resident in a sub-urban Nigeria population..

[33] Araoye MO. Research methodology with statistics for health and social science. Nathadex Publisher, Ilorin. 2003;115–9.

[34] Sowole CA, Sote EO (2007). Early childhood caries: experience in Nigerian children at Lagos. Niger Postgrad Med J..

[35] Olusanya O, Okpere O, Ezimokhai M (1985). The importance of social class in voluntary fertility control in developing country. West Afr J Med..

[36] Folayan MO, Idehen EE, Ufomata D (2003). The effect of sociodemographic factors on dental anxiety in children seen in a suburban Nigerian hospital. Int J Paediatr Dent.

[37] World Health Organisation: *Exclusive breastfeeding*. Retrieved 4^th^ January 2014 from: http://www.who.int/nutrition/topics/exclusive_breastfeeding/en/.

[38] Drury TF, Horowitz AM, Ismail AI, Maertens MA, Rozier RG, Selwitz RH (1999). Diagnosing and reporting early childhood caries for research purpose. Journal of Public Health Dentistry.

[39] World Health Organisation (1997). Oral Health Surveys: basic Methods.

[40] Krapp K: Dental Indices. Encyclopedia of Nursing & Allied Health. Ed. Vol. 2. Gale Cengage. eNotes.com. retrieved 2^nd^ January 2012 from: http://www.enotes.com/dental-indices-reference/.2002.

[41] Greene JC, Vermillion JR (1964). The simplified oral hygiene index. J Am Dent Assoc..

[42] Nunes AM, da Silva AA, Alves CM, Hugo FN, Ribeiro CC (2014). Factors underlying the polarization of early childhood caries within a high-risk population. BMC Public Health..

[43] Folayan MO, Sowole CA, Kola-Jebutu A, Owotade FJ (2012). Risk factors for rampant caries in children from southwestern Nigeria. Afr J Med Med Sci..

[44] Niji R, Arita K, Abe Y, Lucas ME, Nishino M, Mitome M (2010). Maternal age at birth and other risk factors in early childhood caries. Pediatr Dent..

[45] Ola DO, Gambôa ABO, Folayan MO, Marcene W: Family structure, socio-economic position and oral health services utilisation in Nigerian senior secondary school pupils. *Journal of Public Health Dentistry* 2012; Sep 13. doi: 10.1111/j.1752-7325.2012.00362.x. 10.1111/j.1752-7325.2012.00362.x 22970821

[46] Altman DG (2004). Practical statistics for medical research.

[47] Sowole A, Sote E, Folayan M (2007). Dental caries pattern and predisposing oral hygiene related factors in Nigerian preschool children. Eur Arch Paediatr Dent..

[48] U.S. Department of Health and Human Services (2000). Oral health in America: a report of the Surgeon General – executive summary.

[49] Marrs JA, Trumbley S, Malik G (2011). Early childhood caries: determining the risk factors and assessing the prevention strategies for nursing intervention. Pediatr Nurs..

[50] Folayan MO, Kolawole KA, Chukwumah NM, Oyedele T, Agbaje H, Onyejaka N, Oziegbe EO, Oshomoji OV: Effectiveness of the use of caries prevention tools in reducing caries risk in a sub-urban population of children in Nigeria (Abstract 83). Abstract presented at the IAPD Congress, Glasgow, Ireland. July 1^st^ to 4^th^, 2015.

[51] Marinho VCC, Higgins JPT, Logan S, Sheiham A (2009). Fluoride toothpastes for preventing dental caries in children and adolescents (Review).

[52] Tinanoff N, Reisine S (2009). Update on early childhood caries since the Surgeon General's Report. Acad Pediatr..

[53] Davies GM, Blinkhorn FA, Duxbury JT (2001). Caries among 3-year-olds in greater Manchester. Br Dent J..

[54] Szatko F, Wierzbicka M, Dybizbanska E, Struzycka I, Iwanicka-Frankowska E (2004). Oral health of Polish three-year-olds and mothers’ oral health-related knowledge. Community Dent Health..

[55] Harrison R, Wong T, Ewan C, Contreras B, Phung Y (1997). Feeding practices and dental caries in an urban Canadian population of Vietnamese preschool children. ASDC J Dent Child..

[56] Harrison R, White L (1997). A community-based approach to infant and child oral health promotion in a British Columbia First Nations community. Can J Community Dent..

[57] Milnes AR (1996). Description and epidemiology of nursing caries. J Public Health Dent.

[58] Peterson PE: *The world oral health report. Continuous improvement of oral health in the 21st century - the approach of the WHO Global Oral Health Programme*. WHO, Geneva. 2003. Retrieved 31^st^ March 2015 from. http://www.who.int/oral_health/media/en/orh_report03_en.pdf.

[59] Colak H, Dülgergil CT, Dalli M, Hamidi MM (2013). Early childhood caries update: A review of causes, diagnoses, and treatments. J Nat Sci Biol Med..

[60] Folayan MO, Ozeigbe E, Oyedele T, Ola D (2013). Factors limiting dental service utilization by pupils in Ile-Ife, Nigeria. Nigeria Journal of Health Sciences.

[61] ICDAS: *Research*. Retrieved 14^th^ March 2015 from: https://www.icdas.org/research.

[62] Li R, Scanlon KS, Serdula MK (2005). The validity and reliability of maternal recall of breastfeeding practice. Nutr Rev..

[63] Folayan MO, Sofola OO, Oginni AB (2012). Caries incidence in a cohort of primary school students in Lagos State, Nigeria followed up over a 3 years period. Eur Arch Paediatr Dent.

